# Metropolitan National Nursing Association

**Published:** 1891-05-30

**Authors:** 


### Metropolitan and National Nursing Association.

We specially desire to commend, at this time when those who are charitably inclined are making up their minds as to which institution shall receive their gifts, this association, whose object is to provide the sick poor with all the advantages of skilled attendance in their own homes, bringing into many houses not only the benefits derived directly from trained knowledge, but in many instances the first suggestions that such benefits were within the reach of all. To those who visit amongst our poor, the sight of these nurses in their neat bonnet and cloak, and carrying the inevitable black bag (the badge of their mission of usefulness) is as familiar as it is welcome. The following are the names of nursing homes founded by the Committee, or supplied with nurses trained at the Central Home, which will show what a wide scheme of usefulness this Association embraces: Holloway, Paddington, Battersea, Hampstead, Kensington, Newington and Walworth, Chelsea, Windsor, Egham, Westminster, Bishop Auckland, Hertford, Hereford, and Haggerston. During the past year 686 cases were nursed, and the number of visits paid was 20 000 Contributions should be sent to the honorary secretary, the Rev. Dacre Craven, 23, Bloomsbury Square, W.C. Superintendent, Miss Amy Hughes.

